# Semi-automated segmentation of pre-operative low grade gliomas in magnetic resonance imaging

**DOI:** 10.1186/s40644-015-0047-z

**Published:** 2015-08-14

**Authors:** Zeynettin Akkus, Jiri Sedlar, Lucie Coufalova, Panagiotis Korfiatis, Timothy L. Kline, Joshua D. Warner, Jay Agrawal, Bradley J. Erickson

**Affiliations:** Department of Radiology, Mayo Clinic, 200 First St. SW, Rochester, MN 55905 USA; International Clinical Research Center, St. Anne’s University Hospital Brno, Brno, Czech Republic; Neurosurgical Department of 1st Faculty of Medicine of Charles University, Military University Hospital, Prague, Czech Republic

## Abstract

**Background:**

Segmentation of pre-operative low-grade gliomas (LGGs) from magnetic resonance imaging is a crucial step for studying imaging biomarkers. However, segmentation of LGGs is particularly challenging because they rarely enhance after gadolinium administration. Like other gliomas, they have irregular tumor shape, heterogeneous composition, ill-defined tumor boundaries, and limited number of image types. To overcome these challenges we propose a semi-automated segmentation method that relies only on T2-weighted (T2W) and optionally post-contrast T1-weighted (T1W) images.

**Methods:**

First, the user draws a region-of-interest (ROI) that completely encloses the tumor and some normal tissue. Second, a normal brain atlas and post-contrast T1W images are registered to T2W images. Third, the posterior probability of each pixel/voxel belonging to normal and abnormal tissues is calculated based on information derived from the atlas and ROI. Finally, geodesic active contours use the probability map of the tumor to shrink the ROI until optimal tumor boundaries are found. This method was validated against the true segmentation (TS) of 30 LGG patients for both 2D (1 slice) and 3D. The TS was obtained from manual segmentations of three experts using the Simultaneous Truth and Performance Level Estimation (STAPLE) software. Dice and Jaccard indices and other descriptive statistics were computed for the proposed method, as well as the experts’ segmentation versus the TS. We also tested the method with the BraTS datasets, which supply expert segmentations.

**Results and discussion:**

For 2D segmentation vs. TS, the mean Dice index was 0.90 ± 0.06 (standard deviation), sensitivity was 0.92, and specificity was 0.99. For 3D segmentation vs. TS, the mean Dice index was 0.89 ± 0.06, sensitivity was 0.91, and specificity was 0.99. The automated results are comparable with the experts’ manual segmentation results.

**Conclusions:**

We present an accurate, robust, efficient, and reproducible segmentation method for pre-operative LGGs.

## Introduction

Magnetic resonance (MR) imaging is a non-invasive medical imaging technique that provides excellent soft tissue contrast and has become the standard imaging technique for brain tumor diagnosis [1]. Gliomas are the most frequent primary brain tumors [2] originating from glial cells and can be classified into four World Health Organization (WHO) grades (I, II, III, and IV) based on their aggressiveness [3]. Grade I tumors are distinctive in the population (pediatric) and location (often posterior fossa) and constitute a distinct group. The Grade II or ‘Low Grade Gliomas’ (LGGs) are a more challenging group that more frequently affects young adults. LGGs are subdivided based on the microscopic appearance of the tumor into oligodendrogliomas, astrocytomas, and mixed or other gliomas [4]. Compared to high grade gliomas (HGGs), LGGs are less aggressive tumors with better prognosis [4]. In addition, assessment of tumor type and genetic biomarkers (e.g., 1p19q status) may provide valuable information on the response of a given therapy for LGG [4, 5]. Accurate and reproducible segmentation of LGGs is a pre-requisite step for investigating imaging biomarkers that might be related to tumor types and genetic biomarkers.

Manual segmentation of brain tumors is a tedious task with low reproducibility. To avoid this, many semi- and fully-automated segmentation methods have been proposed for brain tumors. Most of these segmentation methods involve classification or clustering approaches [6–10] and have been targeted for HGGs [11]. In addition, most of these approaches require multi-modal datasets for accurate segmentation. Lacking just a single imaging channel renders these segmentation methods unusable. Pre-operative imaging for LGGs is less consistent and all imaging types are not always available; we have found that only pre- or post-contrast T1-weighted (T1W) and T2-weighted (T2W) MR images are consistently available for pre-operative LGGs. This eliminates the use of most classification and clustering approaches for pre-operative LGGs. Edge or region based approaches [12–14] either alone or in combination with classification approaches [15–18], which are based only T2W or T1W images, are more suitable for segmentation of pre-operative LGGs. In some of these approaches [15, 16], normal brain atlases were used to derive prior spatial knowledge. Kaus et al. [15] presented a template-driven classification technique that involves iterative statistical segmentation of LGGs based on T1W MR image intensity values. This method requires manual selection of four points for each tissue type for initialization of the algorithm. An anatomical atlas is used to derive the spatial location of anatomic structures by nonlinear registration of the atlas to each patient’s data. The main limitation of this method is that it fails to properly segment heterogeneous LGGs. Ho et al. [14] proposed a level set evolution method to segment brain tumors. They estimated a tumor probability map from images obtained from subtraction of post- and pre-contrast T1W images and used it as a local guide for a level set snake propagation. Moon et al. [19] and Prastawa et al. [17] proposed a probabilistic tissue classification method based on expectation and maximization (EM) for segmentation of brain tumors. Images obtained from subtraction of post- and pre-contrast T1 weighted images are used for segmentation. However, it is known that not all LGGs enhance on post-contrast MRI [4]. Therefore, these methods may fail for non-enhancing tumor cases. Prastawa et al. [16] extended their previous work by an atlas based segmentation method using T2W images and tested it on a limited data (three tumors). However, tumors show wide variety of intensity characteristics on T2W images. Furthermore, tumor growth can deform brain tissue in great degree and makes use of normal brain atlas difficult for segmentation. Hamamci et al. [12] presented a cellular automata technique combined with graph theoretic methods to segment brain tumors in post-contrast MR images. This method might suffer from heterogeneous tumors that include multi-component boundaries. Harati et al. [13] presented a fully automated tumor segmentation based on fuzzy connectedness on post-contrast T1W images. This method detects tumor seed points automatically based on tumor appearance in post-contrast T1W. Sachdeva et al. [18] proposed a content-based active contour for segmentation of brain tumors in different types of MR images (i.e., pre-contrast T1W, post-contrast T1W, and T2W). The method uses both intensity and texture information within the active contour to overcome segmentation difficulties in intensity based techniques. This method requires an initial contour within the tumor region marked by the user and expands the contour through the edge of the tumor with the content-based active contour. However, having the initial contour or seed point within the tumor and growing that might cause detection of false boundary in heterogeneous tumors.

In our study, we present a combination of classification and region based methods using atlas prior information for segmentation of pre-operative LGGs from T2W alone or T2W plus post-contrast T1W MR images. We chose these two image types because they are nearly universally acquired, even in image-guided biopsy examinations. The purpose of this study was to propose an accurate, reproducible, and semi-automated segmentation method that adapts different input images and satisfies the pre-requisite step for investigation of imaging biomarkers of LGGs.

## Method

**Fig. 1:**
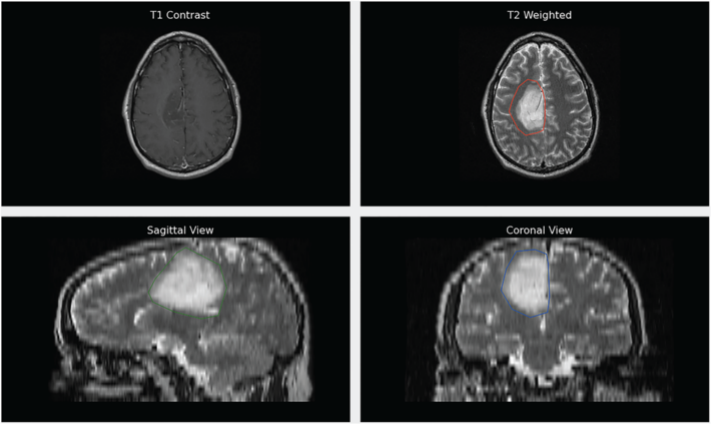
An example of a LGG appearance in post-contrast T1W and T2W images with manually selected tumor ROIs in axial, sagittal, and coronal views of T2W image

We use a combination of two commonly acquired image types, T2W and post-contrast T1W images, as input to our segmentation algorithm. Figure 1 shows an example of LGG image characteristics in post-contrast T1W and T2W images.

**Fig. 2:**
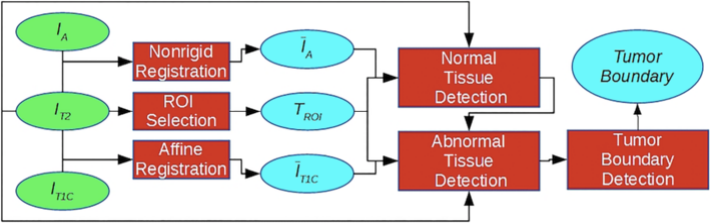
Flowchart of the steps of the algorithm. Inputs (green), operations (red), and outputs (light blue). *I*_*T2*_: T2W image, *I*_*T1C*_: post-contrast T1W image, *I*_*A*_: atlas image, *Ī*_*T1C*_: registered post-contrast T1W image, *Ī*_*A*_: registered atlas image, *T*_*ROI*_: Tumor ROI

Our segmentation algorithm consists of four main steps: ROI creation, image registration, normal brain tissue detection, abnormal brain tissue detection, and tumor boundary detection. A flowchart of the algorithm is shown in Fig. 2.

### ROI creation

The first step of our method is the creation of a region-of-interest (ROI) on the T2W image that completely encloses the tumor and some normal tissue (i.e., white and gray matter). For 2D segmentation, the user draws an ROI on an axial slice of the T2W image. The user must avoid inclusion of cerebrospinal fluid (CSF) in the ROI because CSF and tumors exhibit similar signal on T2W MR images. For 3D segmentation, the user searches for the largest diameter of the tumor on axial views and clicks on the center of the tumor mass. Then, sagittal and coronal views of the tumor for the selected coordinate are displayed and the user draws an ROI that encloses the tumor on the sagittal and coronal views as shown in Fig. 1.

### Image registration

Our segmentation algorithm consists of two image registration tasks. We used the ANTs open source software library for image registration [20, 21]. The first registration task is intra-patient image registration, where post-contrast T1W are registered to T2W images of the same patient. For this purpose, we performed affine registration, which contains linear transformations (e.g. translation, rotation, and scaling), was satisfactory enough to align our intra-patient images.

The second task is registration of a normal brain anatomical atlas to the patient’s T2 image in order to obtain prior normal tissue information. For this purpose, we used the SRI24 atlas [22] that was created using MR images acquired at 3T in a group of 24 normal control subjects. It provides anatomical images (T1W, T2W, and proton density) and probabilistic tissue images (CSF probability, white matter (WM) probability, gray matter (GM) probability, and tissue labels). We used the anatomical T2W atlas image to register the patient’s T2W image in a deformable manner using ANTs diffeomorphic nonrigid registration model. In both of the registration tasks, mutual information [23] was used as the similarity metric.

### Normal brain tissue detection

**Fig. 3:**
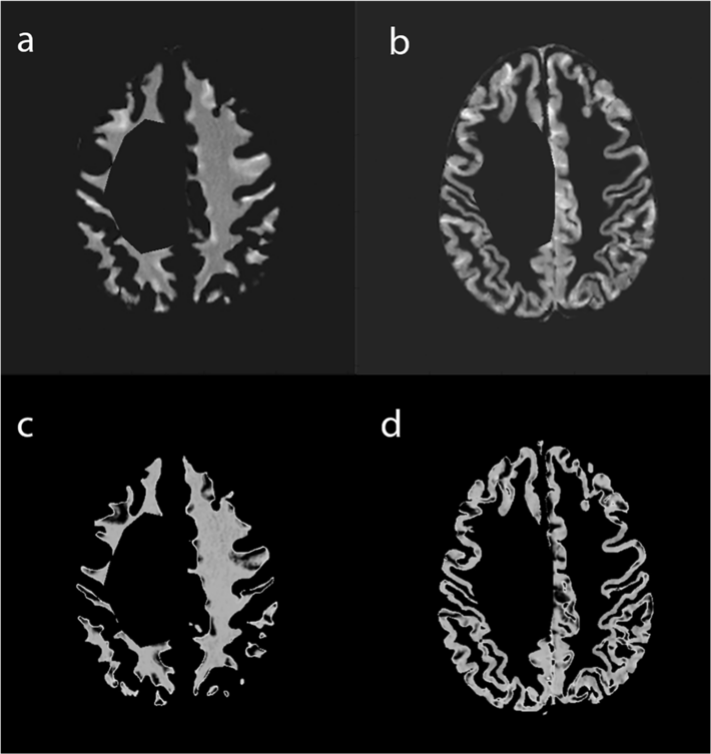
An example of WM and GM after the tumor ROI is masked. WM (**a**) and GM (**b**) before tuning with EM. WM (**c**) and GM (**d**) after tuning with EM

In this step, we obtain a prior normal brain tissue intensity distribution. First, we apply the deformation field, which was obtained through registration of the anatomical T2W atlas and the subject’s T2W image, to the probabilistic atlas images to find corresponding normal brain tissue in the patient’s images (see Fig. 3). Next, we mask the previously defined tumor ROI (*T*_*ROI*_) in the registered probabilistic atlas images to avoid contamination of normal brain tissue with abnormal brain tissue. Afterward, we obtain samples with high atlas probability >0.9 for each of the tissue classes Γ *∈* {CSF, WM, GM} and model the intensity distribution of each class using 2D Gaussian in the joint histogram (*I*_*j *_(***s***) = [Ī_*T*1*C *_(***s***), *I*_*T*2 _(***s***)] where **s** represents the spatial x, y coordinates) of registered post-contrast T1W and T2W images, with parameters *θ*_Γ_ = {*μ*_Γ_^1^, *μ*_Γ_^2^, Σ_Γ_} where *μ*_Γ_^1^ is the intensity mean in *Ī*_*T1C*_, *μ*_Γ_^2^ is the intensity mean in *I*_*T2*_, and Σ_Γ_ is the covariance of intensities of *Ī*_*T1C*_ and *I*_*T2*_. Finally, we refine the intensity distributions of WM and GM by performing the EM algorithm to obtain final distributions (see Fig. 3). In some cases, WM and GM samples will be contaminated with CSF because the registration step might be imperfect. To remove CSF samples from WM and GM, we perform the EM with initialization of two components (*θ*_CSF_ and *θ*_WM_ or *θ*_CSF_ and *θ*_GM_). The EM algorithm maximizes the likelihood iteratively until it converges to a steady state and computes the probability of each pixel belonging to each of the components. In our algorithm, we consider the final WM intensity distribution with parameter *θ*_N_ = *θ*_WM_^*^ as normal brain tissue prior distribution because it generally represents the normal brain tissue around the tumor.

### Abnormal brain tissue detection

In this step, we detect abnormal brain tissue within the ROI, which encloses the tumor and some normal brain tissue, by computing the posterior probability of each pixel in 2D (or voxel in 3D) belonging to normal brain tissue (*N*) and abnormal brain tissue (*A*). First, we construct a joint histogram of the intensities of registered post-contrast T1W (*Ī*_*T1C*_) and T2W images (*I*_*T2*_) within the ROI and define a joint-intensity classifier. We model the joint histogram by a mixture of 2D Gaussians, corresponding to two classes: *ψ* ∈ {*N*, *A*}. Each class *ψ* is modeled by a 2D Gaussian in the joint histogram, with parameters *θ*_*ψ*_ = {*μ*_*ψ*_^1^, *μ*_*ψ*_^2^, Σ_*ψ*_} where *μ*_*ψ*_^1^ is the intensity mean in *Ī*_*T1C*_, *μ*_*ψ*_^2^ is the intensity mean in *I*_*T2*_ and Σ_*ψ*_ is the covariance of intensities of *Ī*_*T1C*_ and *I*_*T2*_. The initialization of parameter *θ*_*N*_ is obtained in the previous step and the initialization of parameter *θ*_*A*_ is obtained from the initial *T*_*ROI*_ distribution. These initial parameters are fed into a k-means clustering algorithm (*k* = 2) and it runs until converging to a steady state. Afterward, we compute the posterior probability *P*(*ψ*_*i*_|*I*_*j*_(*s*)) of each observed pair of intensities (*I*_*j *_(***s***) = [*Ī*_*T*1*C *_(***s***), *I*_*T*2 _(***s***)]) within the *T*_*ROI*_ based on Bayes’ Rule (Eq. 1).

1 $$ P\left({\psi}_i\left|{I}_j(s)\right.\right)=\frac{p\left({I}_j(s)\left|{\psi}_i\right.\right) \Pr \left({\psi}_i\right)}{p\left({I}_j(s)\right)} $$

where p(I_j_(s)|ψ_i_) is the likelihood function, Pr(ψ_i_) is the class prior probability, and p(I_j_(s)) is the marginal likelihood.

**Fig. 4:**
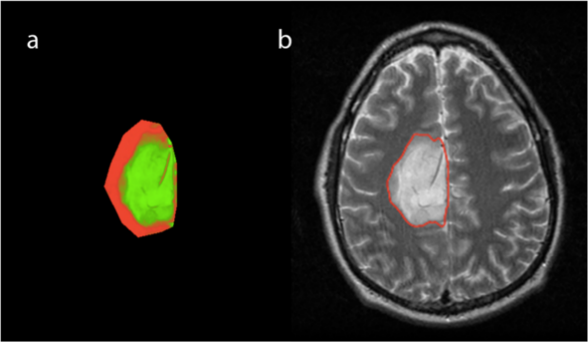
An example of tumor probability map (**a**) shown as an RGB image (Red: Normal tissue, Green: Abnormal tissue, Black: background) for the initial ROI and final segmentation result obtained with GAC (**b**)

The posterior probability of abnormal brain tissue P(ψ_A_|I_j_(s)) > λ, where λ ∈ [0,1], is labeled as final abnormal brain tissue $\left(\widehat{\mathrm{P}}\left({\uppsi}_{\mathrm{A}}\right)\right)$ and assigned to maximum probability (i.e., *p* = 1). The posterior probability map of the patient in Fig. 1 is seen in Fig. 4a.

### Tumor boundary detection

The final step of our algorithm is detection of the tumor boundary by shrinking the initial user defined ROI (*T*_*ROI*_) to the tumor boundary. For this purpose, we used geodesic active contours (GAC) adapted from [24]. The posterior probability of abnormal tissue $\widehat{\mathrm{P}}\left({\uppsi}_{\mathrm{A}}\right)$ is used as the input for the GAC. In the GAC, an energy functional (E(C)) that depends on the image content is assigned to a contour (C) and this energy is minimized until it reaches a steady state (see Eq. 2).

2 $$ E(C)={\displaystyle {\int}_0^{length(C)}\mathit{\mathsf{g}}\left({I}_T\right)}\left(C(s)\right)ds\kern0.1em , where\kern0.5em {I}_T=\widehat{P}\left({\psi}_A\right),\mathit{\mathsf{g}}\left({I}_T\right)=\frac{1}{\sqrt{1+\alpha \left|\nabla {G}_{\sigma}\ast {I}_T\right|}} $$

*where* ∇*G*_*σ*_ ∗ is the first order Gaussian derivative filter with standard deviation *σ*. $\mathit{\mathsf{g}}\left({I}_T\right)$ is low in the edges of the image and high in the other parts of the image. *α* is a constant to adjust gradient strength and empirically set to 10. The minimization of the energy functional is done in the steepest way with the level-set implementation [24]. The initial tumor ROI is considered as the zero level-set and it propagates toward the tumor boundary (see Fig. 4).

### Tumor segmentation in 3D

The segmentation steps for 3D are the same as those defined for the 2D segmentation, with adaptations for a 3D volume, as described here. First, the volume is resampled in the z direction with 1 mm slice thickness using B-spline interpolation to reduce partial volume effects and produce smoother segmentation results. Second, 3D segmentation requires three ROIs as defined in section 2.1—namely, that coronal and sagittal ROIs are also created on the ‘centroid’ slice. From these ROIs, the minimum-bounding box in 3D that encloses the tumor is created. Third, the surface of the bounding box shrinks to the tumor boundary instead of the 2D curve as defined in the previous section. The bounding box may contain detached or attached objects (e.g., a part of the CSF or skull) to the tumor. If separate objects are detected in 3D segmentation results, they are discarded by binary labeling, as the tumor label is known from the ROI drawn on the axial slice. However, if there are objects attached to the tumor (e.g., a tumor close to the ventricles), this requires post-processing to delete or disconnect these objects from the tumor by using a brush tool and updated segmentation results.

### Software implementation and computation

Our LGG segmentation algorithm was implemented in Python programming language. The graphical user interface (GUI) was built in QT (The QT Company Ltd., Digia PLC, Helsinki Finland) designer environment. The image viewer part of the GUI was designed by using the Python matplotlib library and embedded in the QT environment. The software includes several tools such as bias field correction, skull stripping, image registration, image segmentation, and interactive drawing tools.

To perform the experiments, we used a MacBook Air (Apple Computer, Cupertino, CA) with a 1.4 GHz Intel Core i5 processor and 8 GB 1600 MHz DDR3 RAM. The algorithm takes ~1 min to perform intra-patient image registration and, on average, 3 min for atlas registration. Drawing a 2D tumor ROI takes less than 10 s and 3D typically is less than 30 s. The segmentation process itself takes less than 5 s.

### Dataset

Thirty pre-operative LGG patients were randomly selected from our brain tumor patient database at Mayo Clinic Institutional Review Board for this study. Institutional Review Board (IRB) approval was obtained for this study and patient consent was waived. Post-contrast T1W and T2W weighted images were available for all selected patients. All images had dimensions (x,y,z) of 256×256×Z where the number of images (Z) varied from 20 to 64 among patients. The mean voxel size was 0.84 × 0.84 × 4.3 mm^3^ with the Z thickness ranging from 3 to 5 mm.

### Evaluation

In all thirty LGG patients, the axial slice with the largest tumor diameter was visually chosen and manually segmented by three experts (A, L, J) for evaluation of automated segmentation. For this paper, we use the term ‘expert’ to refer to the manual tracings of tumor margins or each person, and below, use the term ‘operator’ to refer to the segmentations resulting from the semi-automated software for each person. The intra-expert and inter-expert variability were measured by computing coefficient of variance (CV). The CV was calculated as follows: *CV*(%) = 100 ⋅ (*SD*_*v*_/*Mean*_*v*_), where *SD*_*v*_ and *Mean*_*v*_ are standard deviation and mean in slice volumes of three manual segmentations. The three manual segmentations were fed into STAPLE (Simultaneous Truth and Performance Level Estimation) software [25] to estimate the true segmentation (TS). The STAPLE algorithm considers a set of segmentations and computes a probabilistic estimate of the TS. The performance of each manual and automated segmentation was measured with the STAPLE algorithm. Automated segmentation was performed in 2D and 3D and was validated against TS for the same slice. For evaluation of results, several metrics were generated such as Dice Index (DI), Jaccard Index (JI), sensitivity $\left(\widehat{p}\right)$, specificity $\left(\widehat{q}\right)$, positive predictive value (PPV), and negative predictive value (NPV) (see Eq. 3).

$$ DI=\frac{2\left|A\cap B\right|}{\left|A\right|+\left|B\right|},JI=\frac{\left|A\cap B\right|}{\left|A\cup B\right|} $$

where A and B are two tumor slice volumes.

3 $$ \widehat{p}=\frac{TP}{TP+FN},\kern0.5em \widehat{q}=\frac{TN}{TN+FP},\kern0.5em PPV=\frac{TP}{TP+FN},\kern0.5em NPV=\frac{TP}{TP+FN} $$

where TP = true positives, TN = true negatives, FN = false negatives, FP = false positives.

We evaluated the intra-operator variability of automated segmentation results by comparing the results of two ROIs drawn by the same operator two weeks apart. For inter-operator variability of automated segmentation, we compared the segmentation results of two different operators using the CV metric.

Our method was also evaluated on a subset of LGG data (25 patients) from BraTS (Brain Tumors Segmentation Challenge, MICCAI 2014). A slice was selected for each patient and automated segmentation was validated against manual ground truth of one expert. We also compared our segmentation results to the segmentation results from an EM algorithm instead of posterior probability.

For selection of parameter λ, the threshold for being classified as abnormal tissue, we divided our dataset into two sets of 15 patients as training and test datasets, and was evaluated for a range of values *λ* *∈* {0.1, 0.2, 0.3, 0.4, 0.5} to select the optimal λ value.

## Results

The intra- and inter-operator variability for automated segmentation is on the same order as intra-expert variability for manual segmentation and is about three times lower than inter-expert variability for manual segmentation as seen in Table 1.

**Table 1:** Intra- and inter-observer variability for manual and automated segmentations

	Manual segmentation		Automated segmentation	
	Intra-expert	Inter-expert	Intra-operator	Inter-operator
CV(%)	3.2 ± 3.1	9.5 ± 4.9	2.3 ± 2.4	3.7 ± 2.5

Units are in percentage (%)

**Fig. 5:**
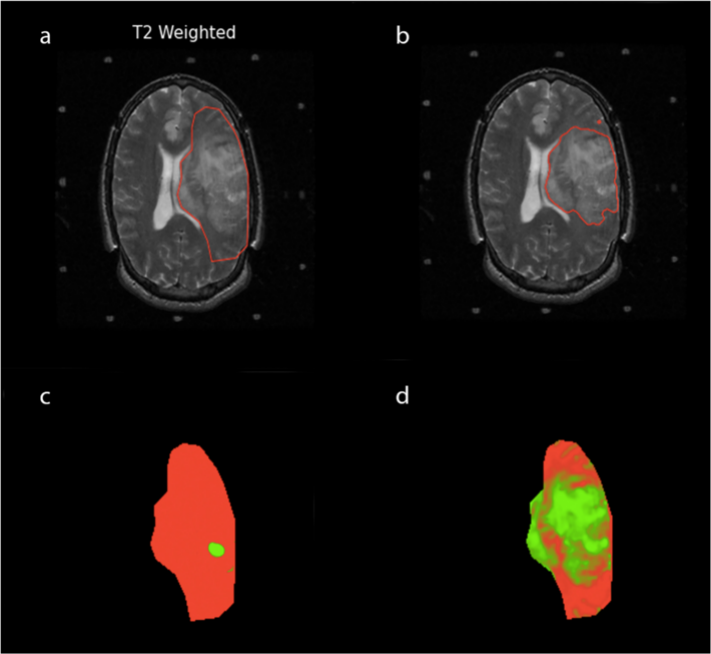
An example of our segmentation result for a heterogeneous tumor. **a** initial ROI. **b** final segmentation with our posterior probability estimation. **c** probability map obtained with EM. **d** probability map obtained with our posterior probability estimation for the initial ROI. Probability maps are shown as an RGB image for the initial ROI. Red: Normal brain tissue. Green: Abnormal brain tissue. Black: Background

**Fig. 6:**
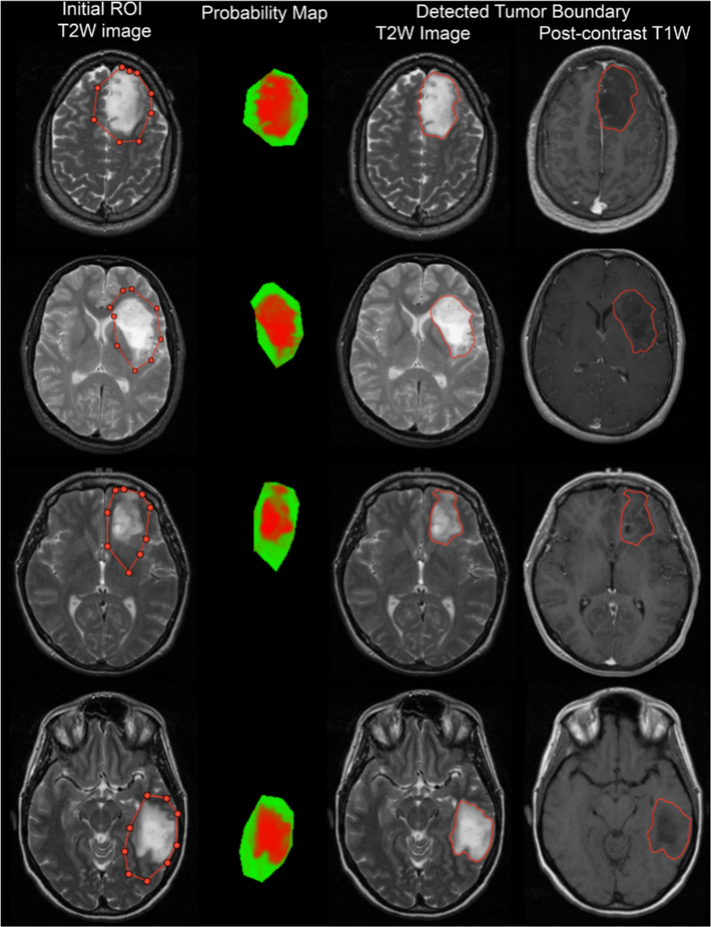
Examples for segmentation result of our method. First column: Initial ROI on T2W images (left-most). Second column: Posterior probability map for ROI. Probability maps are shown as an RGB image for the initial ROI. Red: Normal brain tissue. Green: Abnormal brain tissue. Black: Background. Third and Forth columns: Final tumor boundaries on T2W and post-contrast T1W images

Table 2 shows the performance evaluation of each segmentation compared to the STAPLE-derived TS. Expert A had the highest sensitivity and the highest DI among the experts. Comparing experts L and J, we observe that they have similar DI. However, expert J has higher sensitivity than expert L. This means that expert L comparatively underestimates segmentations and expert J comparatively overestimates segmentations. First segmentations by the first operator (Op1), second segmentations by the first operator (Op1’), segmentations by the second operator (Op2), and segmentations using only T2W images (T2W) have approximately similar sensitivity and DI. 3D segmentation has slightly lower sensitivity compared to 2D segmentation. Automated segmentations have higher sensitivity than expert L but lower sensitivity than experts A and J. DI of automated segmentations are nearly the same order with the DI of experts L and J. Segmentations using EM had lower sensitivity and DI compared to our proposed method. In particular, EM underestimates segmentation in heterogeneous tumors as seen in Fig. 5. Segmentation results of our method for four LGG cases are shown in Fig. 6.

**Table 2:** Performance of all segmentations compared to STAPLE true segmentation

	$\widehat{p}$	$\widehat{q}$	PPV	NPV	DI	JI
A	0.979	0.999	0.965	0.999	0.97 ± 0.05	0.94 ± 0.08
L	0.883	0.998	0.951	0.996	0.91 ± 0.06	0.84 ± 0.09
J	0.967	0.996	0.882	0.999	0.92 ± 0.08	0.86 ± 0.11
Op1	0.922	0.996	0.893	0.997	0.90 ± 0.06	0.82 ± 0.09
Seg3D	0.907	0.996	0.879	0.997	0.89 ± 0.06	0.80 ± 0.09
Op2	0.916	0.997	0.900	0.997	0.90 ± 0.05	0.82 ± 0.08
Op1’	0.914	0.996	0.896	0.997	0.90 ± 0.06	0.82 ± 0.09
T2W	0.920	0.996	0.893	0.997	0.90 ± 0.06	0.82 ± 0.09
EM	0.830	0.996	0.906	0.994	0.84 ± 0.17	0.75 ± 0.18

A, L, J: Experts’ manual segmentations. Op1: First operator’s segmentation. Seg3D: Segmentation in 3D. Op2: Second operator’s segmentation. Op1’: Second segmentation of the first operator. T2W: Segmentation using only T2W images. EM: Segmentation using EM

Table 3 shows the evaluation of the probability threshold (λ) for being abnormal brain tissue. As seen in Table 3, the segmentation results for λ > 0.1 have almost identical DI with only a slight difference. We chose the value λ = 0.3, which gave the best DI in the training set.

**Table 3:** Dice index for training and test set for evaluation of λ parameter

Dice index	λ				
	0.1	0.2	0.3	0.4	0.5
Training set	0.78 ± 0.22	0.88 ± 0.06	0.89 ± 0.06	0.88 ± 0.07	0.88 ± 0.07
Test set	0.78 ± 0.22	0.89 ± 0.06	0.92 ± 0.05	0.92 ± 0.04	0.92 ± 0.03

When evaluating our algorithm with BraTS 2014 Challenge subset data, we obtained DI = 0.85 ± 0.17. This is comparable to the results shown on the BraTS competition website (DI are in the range of 0.74-0.85) [26].

To make a comparison between 3D segmentation results with and without post-processing (PP), average DI and standard deviation is 0.89 ± 0.6 (with PP) vs. 0.87 ± 0.6 (without PP) for one slice validation against STAPLE TS.

## Discussion

We present an accurate and reproducible segmentation method for pre-operative LGGs using T2W or combined T2W and post-contrast T1W images. Our method is semi-automated; robust to heterogeneous composition, irregular tumor shape, and ill-defined tumor boundaries; and adaptive to inputs of different image types. As mentioned before, there have been several studies for automated brain segmentations. However, most of the methods [6–8] require multi-channel datasets and lacking just a single imaging channel renders them useless. These methods cannot be applied to segmenting pre-operative LGGs that have only T2W and post-contrast T1W MR images. Segmentation of single or limited imaging channels would likely require user inputs to increase accuracy of segmentation. As a result, semi-automated methods are used to improve accuracy and narrow the search field. In some studies [14, 17, 19], images acquired by subtraction of post- and pre-contrast T1W images were used for segmentation. However, the vast majority of LGGs do not enhance after administration of intravenous contrast. This will limit the use of contrast enhancement based methods for LGG segmentation. Other studies [16, 18] use a user defined ROI within the tumor as an initialization and grow it to the tumor boundary. However, this may cause failure in heterogeneous tumors, which often contain areas of low signal that confound such methods. To avoid this, we draw an ROI that encloses the tumor and some normal tissue and shrink the ROI through the normal brain tissue to the boundary of abnormal brain tissue.

As seen in Table 1, intra-operator variability is lower than intra-expert variability and inter-operator variability is much smaller than inter-expert variability. In particular, inter-operator variability is almost on the same order with intra-expert variability, which means that our method provides more reproducible segmentation results than manual segmentation by experts with low variability.

As seen in Table 2, performance of our automated methods is better than the performance of one expert and close to the performance of the other two experts. Using only T2W images for segmentation shows slight differences in results compared to using combined T2W and post-contrast T1W images. This shows that there is no significant benefit of using combined T2W and post-contrast T1W images for segmentation in our dataset. However, using combined T2W and post-contrast T1W images might improve segmentation in heterogeneous tumor cases as it provides extra information. The performance of 3D segmentations shows that we can obtain results approaching the better 2D segmentation results. However, 3D segmentation requires additional user interactions for final segmentation results. EM had the worst performance. EM’s relatively poor performance may be attributable to the fact that it underestimates segmentation in heterogeneous tumors (see Fig. 5).

Our semi-automated method results are comparable to fully automated segmentation results in BraTS challenge [26]. Segmentations of pre-operative LGGs lie in the range of fully automated methods - yet offering the potential of segmenting limited datasets that consist of T2W or T2W and post-contrast T1W MR images only.

As indicated previously, the specific aim of our methods is to provide accurate and reproducible segmentations of pre-operative LGGs, relying on T2W and optionally post-contrast T1W images an important pre-requisite step for investigation of imaging biomarkers of LGGs. A limitation of our study is not being able to distinguish between edema and tumor. We consider both as abnormal tissue. Including T2W FLAIR images may allow segmenting edema in another iteration after boundary detection of abnormal brain tissue. Segmentation results in pre-operative LGGs may be useful as initialization for segmentation of post-operative LGGs, which is more difficult due to resection and CSF infiltration. For post-operative LGGs assessment, inclusion of T2W FLAIR will be necessary to distinguish between CSF and abnormal tissue signal. Furthermore, our method can be extended for further assessment of LGG imaging biomarkers such as longitudinal tumor growth rate, cerebral blood volume, and apparent diffusion coefficients by including other imaging modalities which may allow better prognosis for LGGs.

While we did not measure the operator time for our method, it was clearly faster to provide approximate boundaries in the case of 2D, and also faster to provide just 3 ROIs versus tracing every slice for 3D.

## Conclusion

In conclusion, our method provides accurate and reproducible segmentations of pre-operative LGGs, relying on T2W images, with optional T1W MR images. This is an essential step for investigating imaging biomarkers of LGGs and for tumor assessment on follow-up exams.
